# Supplementary material for: Volcano generated tsunami recorded in the near source
Source: Nat Commun. 2024 Feb 27;15:1802. doi: 10.1038/s41467-024-45937-1 (PMC10899579; doi:10.1038/s41467-024-45937-1)
Supplement: Supplementary file 1 — Supplementary Information [file 41467_2024_45937_MOESM1_ESM.pdf]

Supplementary Information for

Volcano generated tsunami recorded in the near source

M. Ripepe* and G. Lacanna

Dipartimento di Scienze della Terra, Università di Firenze, 50121 Florence, Italy

*Correspondence to: maurizio.ripepe@unifi.it

Supplementary Note 1

Video Image Analysis

Visible images are recorded from the camera at LBZ site (Fig. 1, Supplementary Fig. 1f) with focal plane array of 640x480 pixels and collected at a frame rate of 0.5 Hz. The LBZ camera is located at 190 a.s.l (Fig. 1, Supplementary Fig. 1) with a view direction of 215°N and equipped with a FOSCAM camera characterized by focal length of 4 mm and by an field of view of 70° and 75° in horizontal and diagonal directions, respectively. The LBZ images recorded during the pyroclastic flow (Supplementary Fig. 1a-d, Supplementary Movie 1) were georeferenced using IMGRAFT software [*1*] combining the high-resolution Stromboli DEM with the camera parameters (i.e location, view direction, focal length, angle of view and pitch angle). The software is used to project back and forth between pixel and real-world coordinates. We projected the pixel associated to the front of the pyroclastic flow to find the point on the DEM which allows to estimate the width (b) orthogonal to the flow direction and the front velocity u_f (Table I and Supplementary Fig. 1). The pyroclastic flow of 28 August is in direct line of sight with LBZ camera (Supplementary Fig. 1) and in good visibility conditions, this allows to accurately follow step-by-step the front position along the Sciara del Fuoco (Supplementary Fig. 1f).

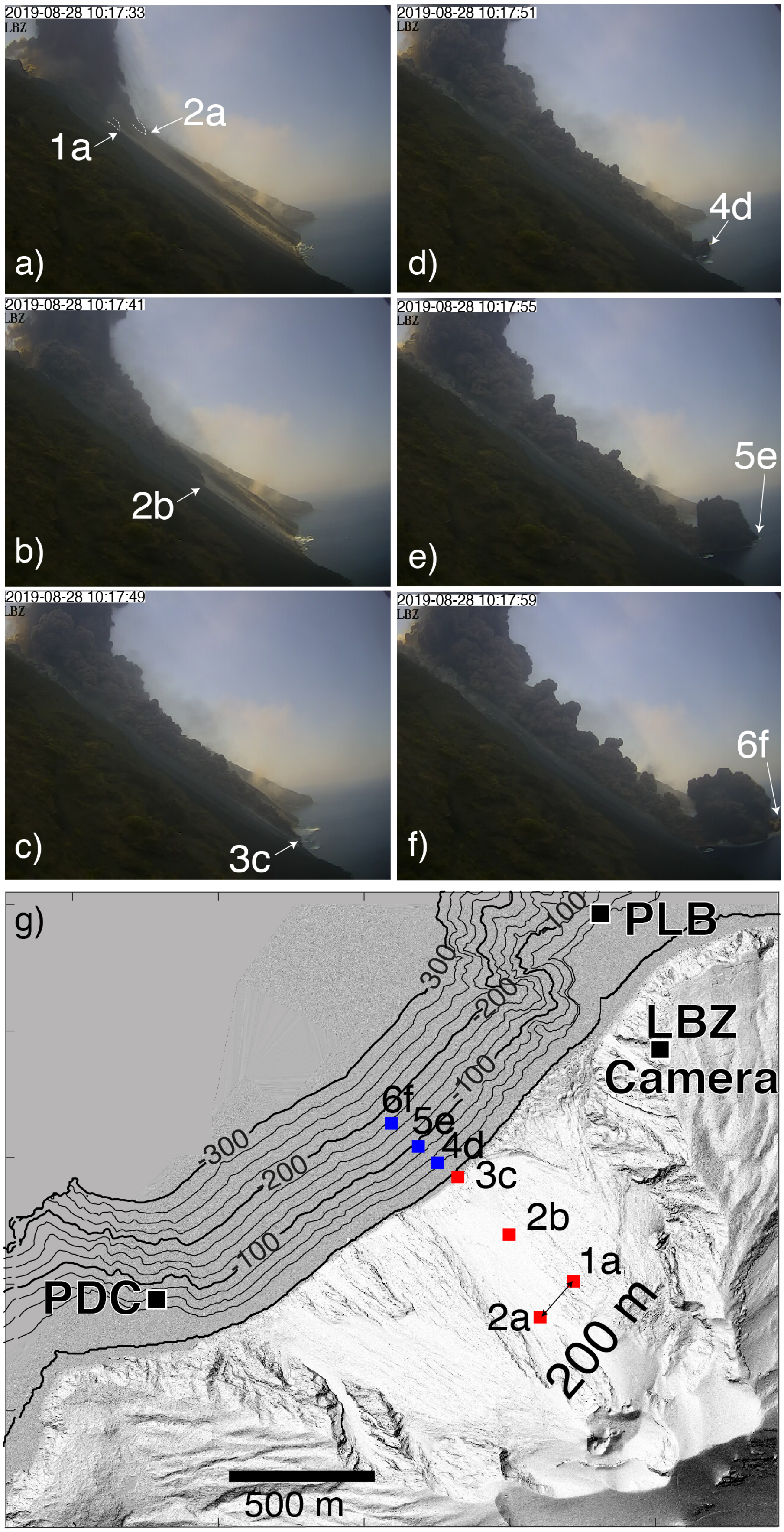

Supplementary Figure 1. a-f) Frames of the video taken by LBZ camera during the 28 August paroxysm. The white arrows indicate the front of pyroclastic flows which are projected on Stromboli DEM in g).

Supplementary Note 2

The elastic beacon, (Resinex Italy srl), is a semi-rigid structure with a 35 m long metallic pipe which is anchored with an anti-torsion steel cable to a concrete block of ~24 tons on the seabed (https://www.resinextrad.com/en/wp-content/uploads/2020/02/Elastic-Beacons.pdf) at 46 m and 50 m depth, respectively for PDC and PLB station (Supplementary Fig. 2). The elastic beacon has a small tower which stands ~10 m above the sea level (Supplementary Fig. 2) and housing data acquisition (Guralp – DM24S6EAMU), radio transmission system (5 GHz 10/100 Mbits and UMTS) and power supply (4 solar panels 110 W). Sensors by IDROMAR (IW05bar) are placed at 14 m along the rigid structure just below the plastic underwater float and at ~50 m depth fixed on the concrete block on the sea bed (Supplementary Fig. 2). Each sensor is measuring the hydrostatic pressure sampled at 125 Hz and the sea temperature at 4 Hz.

Assuming the linear theory [*2*], we consider that pressure is exponentially attenuated by water column height (Supplementary Fig. 2) following the transfer function given by:

$$K(h) = g\,\frac{\cosh(kh)}{\cosh(kH_0)} \qquad (1)$$

where g is the gravity acceleration, k the wave number, h pressure sensor depth and H_0 is the depth of the seabed. This equation allows to calculate the frequency response of the pressure sensors with depth (Supplementary Fig. 2). The sensor at 46 m depth is still sensitive to waves with period above 50 s but strongly reduce by ~87% the periods below 13 s typical of the sea storm.

For sensors deployed at depth larger than 1000 m the attenuation becomes very large also in the period range of 50 – 200 s which is characteristics of tsunami generated by volcano and/or landslides. The depth of ~50 m is then optimal to record tsunami of volcanic origin contributing to reduce by more than 50% the noise generated by the normal sea waves (Supplementary Fig. 2).

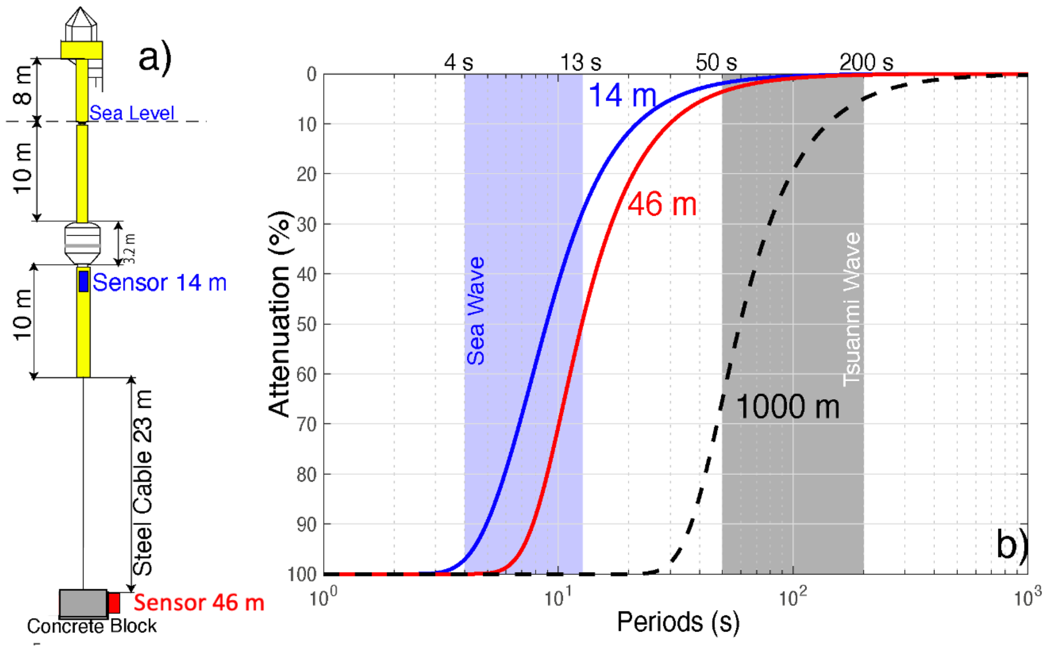

Supplementary Figure 2. a) Sketch of elastic beacon installed at PLB and PDC location redrawn from the blue print of the Resinex Trading s.r.l. b) Attenuation curve for pressure sensor located at 14 m (blue line), 46 m (red line) and 1000 m (dashed black line) b.s.l.

Supplementary Note 3

Tsunami Early- Warning alert system

The tsunami early-warning algorithm is grounded on 4 consecutive steps: 1) spike removal, 2) detrending signal for tide removal, 3) low-pass filtering and finally 4) the STA/LTA ratio algorithm to detect the tsunami wave :

1) Spikes, or sudden high frequency signal, are usually the result of transmission error in the telemetry, disturbance from short electronic glitches or in the case of Stromboli also by fishing activities around the elastic beacons. It is important to detect and eliminate these spikes that can contaminate the record resulting in possible false detection. A method that has proven to be effective in despiking signal is based on the standard deviation σ [*3*]:

$$\lambda_U = \sqrt{2 \log_e n}\ \sigma \tag{2}$$

where n is number of samples considered and λ_U is the expected maximum standard deviation normalized to n data.

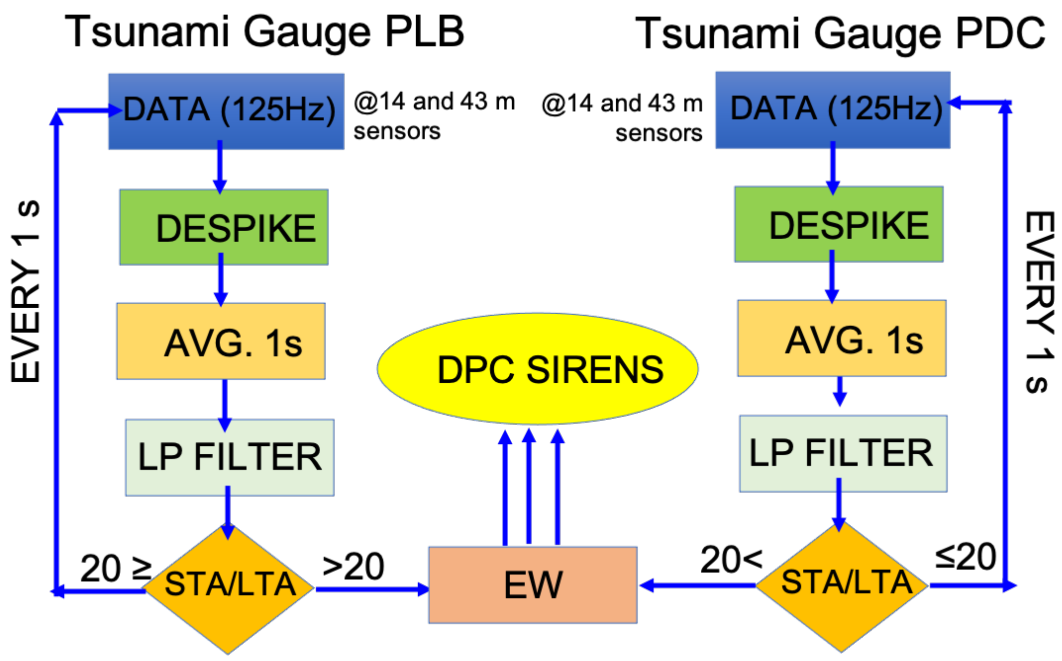

Supplementary Figure 3. Flow diagram of the tsunami early warning at Stromboli

Normally distributed data will lie within λ_U , wheras spikes will be larger than λ_U. We fix the threshold to delete the spikes at $1.5\lambda_U$ [*4*]. Standard deviation is calculated in 1 s time window (*n*=125 samples) shifted by 1 s (with no overlap) and considering different sea condition. This has allowed to fix a threshold $\lambda_U = 0.2$. A logical filter recognizes the spike when $\sigma > 1.5\lambda_U$ and it removes the full one second data interval.

2) If no spikes are detected, the mean value of the 125 samples is considered and the pressure is thus decimated from 125 sampling rate down to 1 sample per second. Data are then linear detrended every second in a time window as large as LTA (4500 samples) in order to remove tidal oscillations from the signal.
3) To filter out the sea waves component, the LTA time series detrended, is convolved with the low-pass Finite Impulse Response (FIR) filter with a cutoff frequency f_c=0.0667 Hz and a transition bandwidth of 0.04 Hz. Time series is extended for edge correction [*5*] with mirror reflection before the convolution with the low-pass FIR filter. Filter preserves the tsunami waveform whereas it reduces strongly the sea wave component, improving the STA/LTA ratio which is two times higher than respect to the unfiltered sea level (Supplementary Fig. 4b).
4) Finally, the STA/LTA ratio is applied to detect the tsunami wave. The STA/LTA ratio [*6*] remains as the most popular method to detect a signal from the noise. The idea behind the STA/LTA ratio method is simple. The ratio is calculated every second as the average energy (or

envelope of the absolute amplitude) of a signal in two different time intervals moving windows, a short-term (STA) and a long-term (LTA) window. The average energy y_t (or envelope function) is estimated by using the Hilbert transform:

$$y_t = \sqrt{\eta^2 + H(\eta)^2} \tag{3}$$

where H denotes Hilbert transform and η the amplitude of the sea level. When the STA/LTA ratio exceeds a predetermined threshold, a detection is declared.

To assure the correct description of the sea state and to reduce the statistical scatter, the LTA window should contain at least 300 waves which considering a sea wave period of 15 s (typical of rough sea state in the southern Mediterranean) corresponds to 4500 s. The selection of the STA window length depends instead strictly on the tsunami wave period and was fixed to 40 s which gives the larger value of ratio STA/LTA at the onset of tsunami for periods ranging between 40 and 200 s. The threshold ratio for the alert at Stromboli was fixed at STA/LTA =20 which provides the best performance also in rough sea conditions and no false detections also during unpredictable malfunctions of pressure sensor.

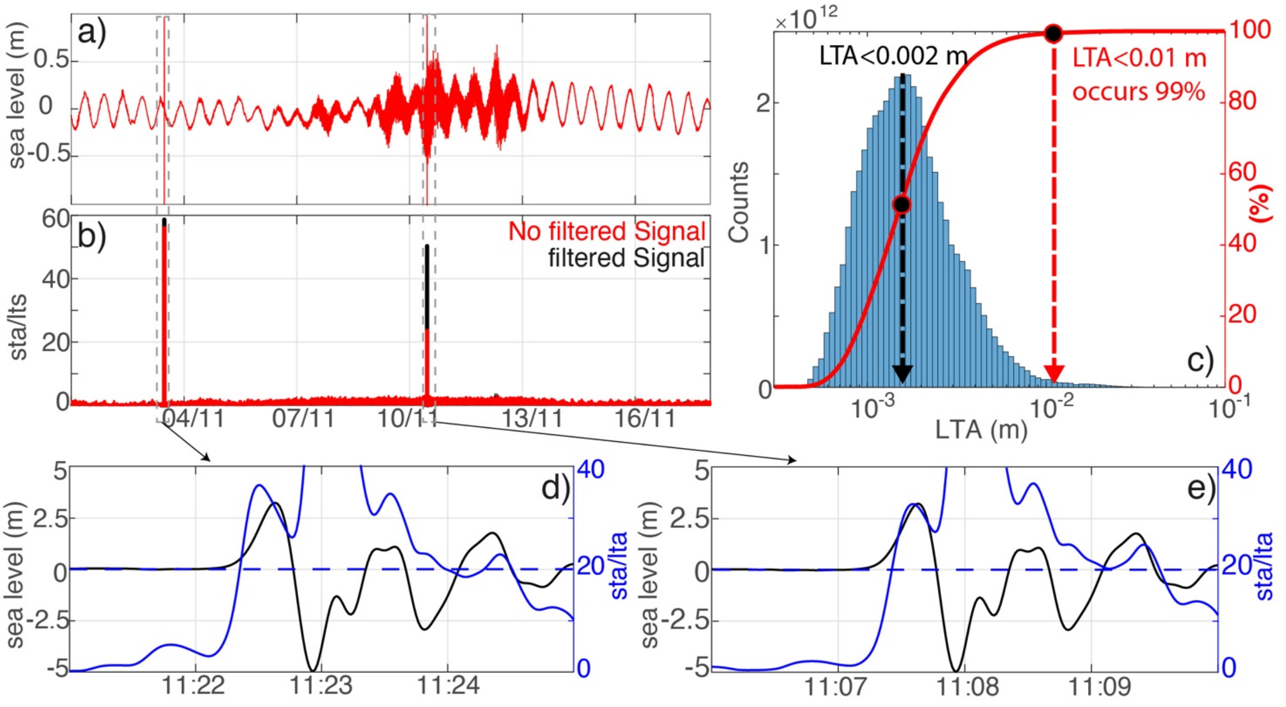

Supplementary Figure 4. Performance of the detection algorithm tested during calm and storm sea conditions. a) Synthetic tsunami waveform was added to sea level data recorded at Stromboli during smooth sea (left gray rectangle) and sea storm (right gray rectangle) conditions. b) STA/LTA applied to the filtered (black line) and

not filtered signal (red line) is above the threshold of 20 in both cases. c) LTA amplitude at PLB calculated for the filtered sea level recorded in the last (2017 – 2022) five years. Considering the STA/LTA=20 threshold, the minimum tsunami amplitude (η =1.57xLTAx20) in the rough sea conditions occurring <1% of the time, with LTA>0.01 m, is ~30 cm. The mean tsunami amplitude in the most common (50% of the time) sea conditions (with LTA ≤0.002 m) is 6 cm. d) STA/LTA ratio (blue line) calculated on the filtered sea level record for a tsunami with amplitude of 5 meters (black line), equivalent to the 30 December 2002 tsunami at Stromboli, assuming smooth sea conditions is detected 7 seconds after the onset and e) 13 seconds in stormy sea conditions.

Supplementary References

1. Messerli A and Grinsted A. (2015). Images Georectification and feature tracking toolbox: ImGRAFT, Geosci. Instrum. Method. Data Syst., 4 23-34, doi:10.5194/gi-4-23-2015
2. Escher J. and T. Schlurmann. On the Recovery of the free surface from the pressure within periodic traveling water waves. *Journal of Nonlinear Mathematical Physics*, **15,** 2, 50-57 (2008).
3. Donoho, D. L., and Johnstone, I. M. (1994). “Ideal spatial adaptation by wavelet shrinkage.” *Biometrika*, **81** (3), 425–455.
4. Goring D.G. Extracting long waves from tide-gauge records. *Journal of Waterway, port coastal, and ocean engineering*. **134**, 5, September 1, 2008.
5. Wah, B. W., and M. Qian (2002), Constrained formulations and algorithms for stock-price predictions using recurrent FIR neural networks, in Proceedings of the 18th National Conference on artificial intelligence (AAAI-2002), Edmonton, Alberta, Canada edited by R. Dechter, M. Kearns, and R. Sutton, The AAAI Press, Menlo Park, Calif.
6. Allen, R. V. (1978). Automatic earthquake recognition and timing fromsingle traces. Bulletin of the Seismological Society of America 68,1,521–1,532.
