# Supplementary material for: Volcano generated tsunami recorded in the near source
Source: Nat Commun. 2024 Feb 27;15:1802. doi: 10.1038/s41467-024-45937-1 (PMC10899579; doi:10.1038/s41467-024-45937-1)
Supplement: Supplementary file 3 — Description of Additional Supplementary Files [file 41467_2024_45937_MOESM3_ESM.pdf]

Description of Additional Supplementary Files

File Name: Supplementary Data 1
Description: The .zip folder contains the four tsunamis' waveforms shown in Figure 3 and sampled at 125Hz. The data of the four waveforms are in Matlab format, the name of each file indicates the tsunami gauge and the day of record. Each matlab file contains two variables: h (sea level elevation in meters) and time (vector time in UTC).

File Name: Supplementary Movie 1
Description: Video of the pyroclastic flow generated by the 3 July 2019, paroxysm taken by the permanent visible camera (LBZ) located at Punta Labronzo (Supplementary Fig. 1g) accelerated 10 times and synchronized with the record of the tsunami at the two gauges b) PDC and c) PLB. Sea level raw data (red signals) are processed following the procedure described in Supplementary Note 3 both for PDC (blue signal in b) and PLB (black signal in c). d) STA/LTA ratio calculated on the processed signal recorded at PDC (blue line) and PLB (black line). Early warning is automatically issued when the STA/LTA ratio at both tsunami gauges (PDC and PLB) reaches the pre-defined threshold of 20 (dashed red line). The background colour in the STA/LTA plot (d) is GREEN when the ratio for both signals are below the threshold, is ORANGE if only one signal reaches the threshold and becomes RED when both signals are above the threshold indicating that a tsunami has been detected. The signal at 14:45:50 is the pressure wave generated by the explosion and recorded at the sensors located at the sea bed (~46 m). In the video is visible a second pyroclastic flow which reaches the sea at 14:46:20 UTC.

File Name: Supplementary Movie 2
Description: Tsunami wave simulation used to estimate the numerical arrival time. This movie represents one case out of the 114 numerical simulations used to estimate the arrival time at PDC and PLB elastic beacon from a source position located at each of 19x6 nodes along the shoreline and offshore the Sciara del Fuoco (see Fig. 4).
